# Cl Anion-Dependent Mg-ATPase

**DOI:** 10.1007/s00232-012-9423-9

**Published:** 2012-03-08

**Authors:** Sopio Dzneladze, Leila Tsakadze, Marina Leladze, Zurab Kometiani

**Affiliations:** Beritashvili Institute of Physiology, 14 Gotua str, 0160 Tbilisi, Georgia

**Keywords:** Plasma membrane, P-type ATPase, Transport-ATPase, Anion dependence ATPase (Cl-ATPase) free ligand, Molecular mechanism

## Abstract

We studied, in the rat brain, the synaptosomal and microsomal membrane fractions of Cl^−^ ion-activated, Mg^2+^-dependent ATPase, satisfying the necessary kinetic peculiarities of transport ATPases, by a novel method of kinetic analysis of the multisite enzyme systems: (1) the [Mg-ATP] complex constitutes the substrate of the enzymic reaction; (2) the V = f(Cl^−^) dependence-reflecting curve is bell-shaped; (3) substrate dependence, V = f(S), curves at a constant concentration of free ligands (Mg_f_, ATP_f_, Cl^−^); (4) as known from the literature, in the process of reaction a phosphorylated intermediate is formed (Gerencser, Crit Rev Biochem Mol Biol 31:303–337, 1996). We report on the Cl-ATPase molecular mechanism and its place in the “P-type ATPase” classification.

## Introduction

Anion-dependent, Mg^2+^-activated ATP hydrolysis, namely, HCO_3_^−^ and activation by Cl^−^ ions, is known from the literature. The activity has been mainly identified in bacteria and eukaryotes, some organs of *Aplysia californica* and toad (*Bufo bufo*) such as the epithelial tissue basolateral membrane of the small intestine (Gerencser and Dept 2003), membrane fraction of freshwater eel fins (Bornancin et al. 1977), the plasma membrane of *Aplysia* vesicles and rat pancreatic canals (Zhao et al. 1994), membrane vesicles of rat brain (Gerencser 1996), liver plasma membrane cells (Izutsu and Siegel 1980) and brain plasma membrane cells (Gerencser and Dept 2003).

The asymmetric distribution of anions (mainly HCO_3_^−^ and Cl^−^) in the membrane (out ≫ in) accounts for their passive transport down the concentration gradient, whose reverse system appears to be an active transport mechanism. Transport ATPases are known to have a particularly important role in cell functioning. Providing the asymmetric arrangement of cations in the membrane at the expense of ATP hydrolysis, they represent a complex biological mechanism.

Among the transport ATPases worth mentioning are the “P-type” ATPases, which possess a phosphorylated intermediate and accomplish a two-step catalysis of phosphorylation and dephosphorylation with the participation of the Mg-ATP complex.

P-type ATPases belong to the plasma membrane ATPases, such as Na, K-ATPase, H-ATPase, K, H-ATPase and bivalent cation-activated ATPases. The ATP hydrolysis activation by anions can also be attributed to P-type ATPases.

The goal of the present work was to identify Cl-ATPase activity in rat brain plasma membrane fractions, to study the enzyme molecular mechanism using the method of composite geometric curve shape analysis and to determine its place in the general classification of ATPases.

## Materials and Methods

Sections of albino rat brain from different membranes of both sexes, weighing 200–300 g, served as the experimental material. Sections were obtained via osmotic shock to the synaptosomes at 0.9–1.2 M sucrose (Kometiani 1982), as well as fractions of microsomes (0.32 M sucrose). The preparations were washed in 2.5 mM EGTA and 2.5 mM EDTA solutions.

ATPase activity was determined by a volume of liberated inorganic phosphorus (P_i_) (Kazanov and Maslova 1980), and protein was determined according to the method of Lowry et al. (1951). ATPase activity was represented as micromoles of P_i_ per hour, with milligram protein units. Reagent medium always contained 30 mM Tris–Malate (pH 7.65), 0.4 mM EGTA, 0.2 mM ouabain and 0.3 mM ethacrynic acid (the specific inhibitor of Cl-ATPase (Gassnez and Komnick 1981; Tanaka et al. 1986). Concentrations of other ligands in incubation solution are given in the text.

Cl-ATPase was measured as the difference between Cl^−^-containing incubation and ethacrynic acid-containing media. Experimental data were processed statistically.

In the Cl-ATPase study we applied kinetic analysis of multisited enzyme systems (Kometiani et al. 1984), which is a single method used from kinetic investigation of multisited enzyme systems.

Concentration estimates of free ATP_f_, Mg_f_^2+^ and Mg-ATP complex were made by application of the following equations:

$$ \left[ {{\text{ATP}}_{\text{f}} } \right] \cdot \left[ {{\text{Mg}}_{\text{f}}^{ 2+ } } \right] = \left[ {{\text{Mg-}}{\text{ATP}}} \right] \cdot K_{\text{Mg}} $$

$$ \sum {\text{Mg}}^{ 2+ } = \left[ {{\text{Mg}}_{\text{f}}^{2+} } \right] \, + \, \left[ {{\text{Mg-}}{\text{ATP}}} \right] $$

$$ \sum {\text{ATP }} = \left[ {{\text{ATP}}_{\text{f}} } \right] + \left[ {{\text{Mg-}}{\text{ATP}}} \right] $$

where *K*_Mg_ is the dissociation constant of the Mg-ATP complex (0.0603 mM).

To analyze the experimental curves, the method of kinetic analysis of multisited enzyme systems was applied. This kinetic method of complex geometric shape curves was used to establish a “minimal model” for the enzyme system. The reaction velocity of the Cl-ATPase enzyme system is a function of at least three physiological ligands, Mg-ATP, Mg_f_^2+^ and ATP_f_, each of which may exert on the enzyme an activating or inhibiting action. To analyze the initial velocity of an enzymatic reaction, it is required to obtain V = *f*([Mg-ATP], [ATP] [Mg_f_^2+^]) as a function of one variable, when the values of other ligands are constant. Therefore, in the experiment the concentrations of the mentioned ligands were chosen so that the enzyme reaction velocity was actually represented by a one-variable ligand function. In particular, in each experiment the concentrations of three ligands represented constant quantities. Then, in each particular case, the conditions of the reaction being invariable and the enzyme functional unit structure being in a steady state, the initial velocity would be a one-variable function and would be described by the following analytical formula:

$$ V = e_{ 0} \frac{{x^{\text{n}} \sum\nolimits_{i = 0}^{p} {\alpha_{\text{i}} x^{\text{i}} } }}{{\sum\nolimits_{i = 0}^{S} {\beta_{\text{i}} x^{\text{i}} } }};\,S = n + m + p $$

where $\alpha_{\text{i}}$ and $\beta_{\text{i}}$ are the sum of products of individual velocity coefficients and steady-state ligand concentrations; X is a variable ligand concentration; $e_{ 0}$ is the enzyme overall concentration; and *n, m* and *p* represent power parameters and are positive integers: *n* is the number of sites for essential activators, *m* is the number of sites assigned for full-effect inhibitors and *p* is the number of sites for partial-effect modifiers. To determine numerical values for the parameters *n, m* and *p*, a special computer program was used (Kometiani 2007).

## Results

From primary evidence it follows that ATP hydrolysis with Cl^−^ participation proceeds only in Mg^2+^ medium. When Mg^2+^ = 0, activation by Cl^−^ does not manifest itself. Consequently, Cl-ATPase activity implies Cl^−^ anion Mg^2+^-activated ATP hydrolysis.

**Fig. 1:**
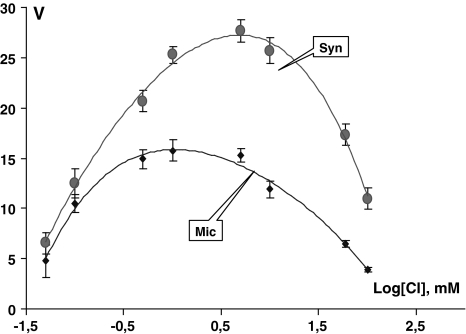
Dependence of Cl-ATPase activity (V) on Cl ion concentration (decimal logarithms) in synaptic and microsomal fraction. Protein: 0.022 mg/ml, ATP = 2 mM, Tris–Malate = 30 mM (pH 7.7), ouabain = 0.2 mM

Figure 1 shows the relation of Mg^2+^-dependent ATP hydrolysis with Cl^−^ ion concentration in synaptic membrane and microsomal fractions. The shape of the V = f(Cl^−^) curve is similar in both cases. Only the specific activity of the enzyme is altered. Synaptic membranes are characterized by a high Cl-ATPase activity compared to microsomes. Analysis of the curve’s geometric shape clearly reveals its bell shape, with ascending and descending phases of Cl^−^ concentration dependence.

Study of Cl^−^ ion activity mechanisms on Cl-ATPase has revealed that when [Cl^−^] < 10 mM the enzyme activity dependence on a variable ligand is increasing. With a further increase of Cl^−^ concentration the enzyme activity is reduced and approximates zero. For the transport to be effected, at the very beginning the enzyme with high affinity should bind an ion, whereas after the transport has been effected, due to the decay of ion affinity, the enzyme should get released from that ion. Such a geometric shape of the kinetic V = f(Cl^−^) curve is certainly specific to all Tr-ATPases and is a necessary but insufficient condition for the identification of Tr-ATPase system.

Qualitative conversion results in linearization of the function when *r* = 1 (*r* is a power index whose numerical value is determined by the curve’s shape; when the function has a horizontal asymptote, *r* = *n*). This means that each molecule of Cl-ATPase has one ligand-binding site for Cl^−^ as for an essential activator (i.e., *n* = 1). To determine the number of sites for Cl^−^ as a full inhibitor, a high variable concentration of Cl^−^ was taken ([Cl] ≥ 40 mM). Linearization of function was achieved when *r* = 1, i.e., number of sites for Cl^−^ as a full inhibitor, *m* = 1.

**Fig. 2:**
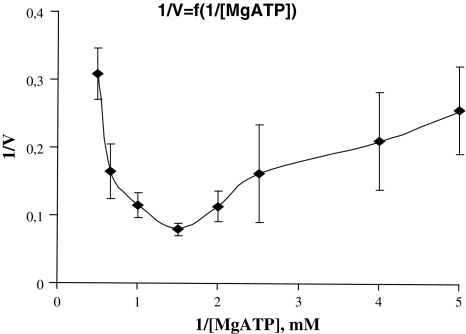
Dependence of Cl-ATPase upon substrate (S = Mg-ATP) contentration, in double inverse values, when Mg_f_ = ATP_f_

Figure 2 represents in double reciprocal values the Cl-ATPase activity dependence on Mg-ATP concentration. In both cases 1/V = f(1/Mg-ATP) function has an asymptote at high values. At medium Mg-ATP concentrations the function has turning and inflexion points, while at small values the enzyme system undergoes inhibition. At high values (with extremely small Mg-ATP concentration) the linearity of 1/V = f(1/Mg-ATP) function is a necessary and sufficient condition for maintaining that the [Mg-ATP] complex represents a true substrate for the enzyme system (Kometiani 1982).

**Fig. 3:**
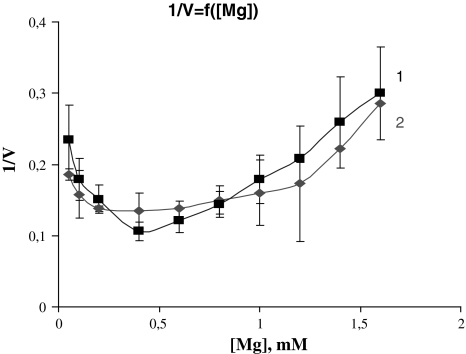
Dependence of Cl-ATPase activity upon Mg_f_ concentration at fixed substrate concentrations (S_1_ = 0.5 mM, S_2_ = 1.5 mM). Microsomal fraction protein = 0.025 mg/ml

Free ligands (Mg_f_ and ATP_f_) are known to be modifiers of transport ATPases (for Na, K-ATPase, in particular). The effect of the given ligands on Cl-ATPase was studied at various fixed substrate concentrations. Figure 3 shows the pattern of ATPase activity change from Mg_f_ concentration under conditions of various fixed substrate concentration (0.5, 1.5 mM).

As is evident from the figure, at low Mg-ATP fixed concentration the curve has a simple concave shape. At a high fixed concentration the curve has a composite geometric shape: at modest values one can see activation of the enzyme system, while at high concentrations the enzyme system is inhibited. Therefore, in the 0.2–1.2 mM range of Mg, enzyme activity is homogeneous and a “plateau” is seen on the curve (Fig. 2).

A similar dependence was observed upon studying V = f(ATP_f_) when S = const. The inhibition pattern by free ligands (Mg_f_ and ATP_f_) appeared to be qualitatively identical.

**Fig. 4:**
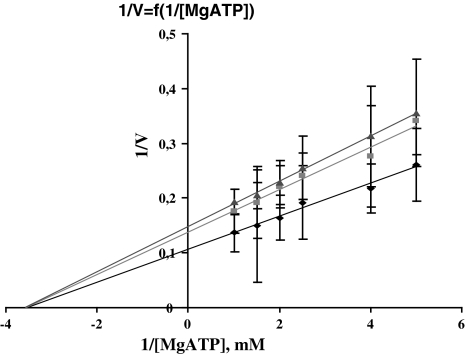
Dependence of Cl-ATPase activity upon substrate concentration on double inverse values at different fixed Mg_f_ concentrations: 0.2, 0.5, 1.2 mM

Figure 4 shows the Cl-ATPase activity dependence on substrate concentration, in double reciprocal coordinates, at various Mg_f_ fixed concentrations. The substrate experimental concentrations were chosen so that V = f(S) had a rectilinear dependence at small concentrations. Under these conditions, with a rise in [Mg_f_], the slope and intercept increase; the lines intersect on the abscissa, which indicates that the enzyme affinity for the substrate does not alter at various fixed Mg_f_ concentrations; the reaction maximum velocity increases in parallel with Mg_f_ concentration rise, within the concentration range; and a further rise in Mg_f_ concentration (>0.8 mM) causes a decline in activity.

**Fig. 5:**
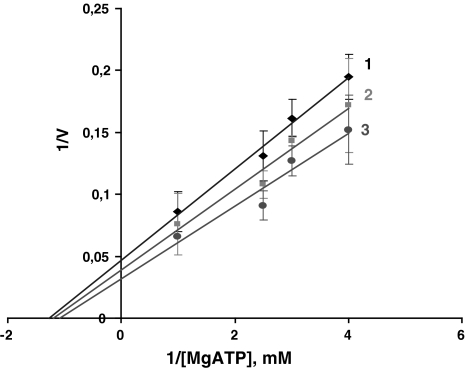
Dependence of Cl-ATPase activity on substrate concentration on double inverse values at different fixed Cl^−^ concentrations: 5, 10, 15 mM

Figure 5 represents Cl-ATPase activity dependence on Cl^−^ ions (x) at different fixed substrate concentrations in double-reciprocal values. The 1/V = f(1/x) function is a straight line and has an asymptote; i.e., Cl^−^ ions are activators of Cl-ATPase. Calculation of regression coefficients and determination of line intersection points allows us to ascertain the character of modifier (Cl^−^ ions in particular) activity for the enzyme system. Computation of regression coefficients for asymptotes revealed that this parameter is identical at various substrate fixed concentrations; i.e., linear functions intersect at one point on the abscissa. And this indicates that the enzyme affinity for the substrate remains unchanged with a Cl^−^ concentration change. Binding of the substrate and Cl^−^ ion is mediated by a randomized mechanism.

## Discussion

The “P-type” cations appear to be multisited, complex enzyme systems. Less known is the role and place in the general classification of anionic ATPases (Cl-ATPase, in particular). To determine this, study of the molecular mechanism of its activity is necessary. First of all, a question is raised as to whether Cl-ATPase belongs to the transport ATPase system or not. The principal demands posed to the transport ATPases are known from the literature (Kometiani and Nozadze 2007). Among them, the most important is to define the shape of the curve reflecting the transportable ion-dependent velocity of V = f(Cl^−^) and the Mg-ATP complex as a true substrate.

For all transport ATPases, the curve reflecting the enzyme reaction velocity dependence on the transportable ion concentration has a bell shape, where the ascending phase corresponds to the ion binding process and the descending one corresponds to its release. According to our data, this condition is satisfied for Cl-ATPase only in the case when the reaction proceeds against the background of Mg^2+^ ions.

From our findings it emerges that Cl^−^ ion-activated, Mg^2+^-dependent ATPase satisfies the essential kinetic peculiarities of transport ATPases:

1. The curve for V = f(Cl^−^) dependence has a bell shape (Fig. 1).
2. The optimal regimen for the system to work provides for the inevitable existence of the [Mg-ATP] complex (Figs. 2,  3).
3. In the course of the catalytic reaction a phosphorylated intermediate is formed (Gerencser 1996).

The rectilinearity of the 1/V = f(1/Mg-ATP) function at small Mg-ATP concentrations (Fig. 2) indicates that the number of sites for Mg-ATP as an essential activator is *n* = 1. If we take into account that Cl-ATPase activity manifests only in the presence of Mg^2+^ and Mg-ATP is an essential activator for the enzyme system, it may be said that Mg-ATP constitutes a substrate for the Cl-ATPase system. It is remarkable that at high concentrations of Mg-ATP its number of sites assigned for full inhibitors is *m* = 1. Under these conditions, on the 1/V = f(1/Mg-ATP) curve at medium values the existence of turning and inflexion points indicates that the minimal number of sites for Mg-ATP as for the partial effect modifiers is *P* ≥ 1. Since in the case of other P-type ATPases, for Na, K-ATPase in particular, it is known that for each subunit of the enzyme there is one substrate binding site, it can be assumed that for Cl-ATPase as well the functional unit is minimum a dimmer with two identical subunits.

At various Mg^2+^ fixed concentrations, analysis of V = f(1/S) revealed its modifier nature as having an enzyme system-inhibiting effect. The result given in Fig. 4 together with the above-mentioned may be considered as verification that Mg^2+^ is a component of Cl-ATPase substrate, though it is more complex in nature. On one functional unit of the Cl-ATPase molecule there may be a special binding site where Mg^2+^ may display its potent modifying nature. The straight line intersection at the abscissa (Fig. 4) indicates that for Mg-ATP Mg is an inhibitor with invariable affinity and Mg^2+^-bound forms of the enzyme have no catalytic ability.

By analysis of the straight line arrangement (Fig. 5) reflecting substrate concentration dependence of Cl-ATPase in double-reciprocal coordinates at varying fixed Cl^−^ concentrations, a conclusion is drawn that Cl^−^ ions are activators of the enzyme system that stimulate preferential activity of the partial effect sites. Cl^−^ is at the same time a modifier of the system. By increasing its concentration, activation passes into inhibition. Similar alignment is possible only in the case of a multisited enzyme system and depends on the parameters resulting from multisitedness.

Thus, Cl^−^ ions must belong to the group of modifiers whose activity results in reduction of the slope of rectilinear function, though the ordinate axis intersection point is unchanged, which is the necessary condition for specific modifiers. This means that Cl^−^ ions are specific effect activators.

Free ligands (Mg_f_ and ATP_f_) are the system’s modifiers, with activating and inhibiting effects, with invariable affinity for the substrate. As to Cl^−^ ions, they are a specific type of modifier with invariable affinity for the substrate. Thus, it may be concluded that all P-type transport ATPases, cations as well as anions, are characterized by a similar molecular mechanism.

The mechanism of dead-end branch formation has been studied in the Na, K-ATPase reaction. During the ATPase reaction, not only the Mg-ATP complex formed in the solution but also the one set up on the enzyme molecule is hydrolyzed. For this to be realized the necessary condition is binding in the enzyme active center, first of ATP and then of Mg^2+^. In case this process takes place in reverse (first Mg^2+^, then ATP), a “dead-end” complex is formed.

In accordance with the data reported (Robinson and Flasher 1979; Kometiani et al. 1984), the presence of only one catalytic site per α-subunit has been identified for the Na, K-ATPase system. Therefore, the molecule has a dimeric nature with two identical subunits, 2(αβ), that must be a universal system for all P-type transport ATPases.

Study of the interrelation of membrane Cl-ATPase with other ATPases will assist to some extent in deciphering the mechanism and function of its activity. To this end, we have reveled anion interaction in the membrane in ATP hydrolysis. In the living cell an excessive amount of anions was found on the external surface of the membrane compared to the internal one. This fact determines their movement within a concentration gradient from outward to inward. There is an indication in the literature that there may also be cotransport of Cl^−^ and other anions at the expense of ATP hydrolyzing energy (cotransport of Cl^−^/HCO_3_^−^ in particular). Cl^−^ is directed from inside the cell, in contrast to HCO_3_^−^. The process may be due to the existence of an Mg-nondependent HCO_3_-ATPase in the plasma membrane (Tsakadze et al. 2007), whose substrate (as distinct from Mg-dependent HCO_3_-ATPase) is only free ATP. This ATPase is likely to be attributed to the so-called Ecto-ATPase group, which function on the external side of the membrane and do not require Mg ions.

## Conclusion

In conclusion, it can be said that the study of Cl-ATPase revealed an interesting analogy of transport ATPases with general kinetic peculiarities. Cl-ATPase, like the P-type ATPases, is a transport system which carries out active movement of Cl^−^ anions from inward to outward of the synaptosomal and microsomal membranes. The kinetic parameters of both membranes are identical. On the basis of the evidence and theoretical calculation, we suggest a scheme for the operation of the Cl-ATPase system.

## References

[CR1] Bornancin M, De Renzis G, Maetz I (1977). Branchial Cl transport, anion stimulated ATPase and acid–base balance in *Anguilla anguilla* adapted to freshwater: effects of hyperoxia. J Comp Physiol B Biochem Syst Environ Physiol.

[CR2] Gassnez D, Komnick H (1981). Inhibition of a Cl^−^/HCO_3_-ATPase in the avian salt gland by furosemide and ethacrynic acid. Cell Biol Int Rep.

[CR3] Gerencser GA (1996). The chloride pump: a Cl^−^ translocating P-type ATPase. Crit Rev Biochem Mol Biol.

[CR4] Gerencser GA, Dept ZI (2003). Existence and nature of the chloride pump. Comp Biochem Physiol A.

[CR5] Izutsu KT, Siegel IA (1980). A microsomal HCO_3_^−^-stimulated ATPase in the rat liver plasma membrane cells. Biocemistry.

[CR6] Kazanov A, Maslova M (1980). Peculiarities of detergent-induced activation of the brain Na, K-ATPase in vertebrates. Zh Evol Biokhim Fiziol.

[CR7] Kometiani Z (1982). Method for the analysis of kinetic curves for multisite enzyme systems. Bull Ga Acad Sci.

[CR8] Kometiani Z (2007). Methods for kinetic analysis of multi-sited enzyme systems.

[CR9] Kometiani Z, Nozadze E (2007). Kinetic singularities of transport ATPases. Bull Ga Acad Sci.

[CR10] Kometiani Z, Tsakadze L, Jariashvili T (1984). Functional significance of the effects of neurotransmitters on the Na, K-ATPase system. J Neurochem.

[CR11] Lowry OH, Rosebroygh NL, Fazz AU, Randall RY (1951). Protein measurement with the folin phenol reagent. J Biol Chem.

[CR12] Robinson ID, Flasher MS (1979). The (Na^+^, K^+^) activated ATPase. Biochem Biophys Acta.

[CR13] Tanaka T, Inagaki C, Matsuda K, Takaori S (1986). Characteristics of ethacrynic acid highly sensitive Mg^2+^-ATPase in microsomal fractions of the rat brain: functional molecular size, inhibition by SITS and stimulation by Cl^−^. Jpn J Pharmacol.

[CR14] Tsakadze L, Dzneladze S, Kometiani Z (2007). Mg-independent HCO_3_-ATPase. Ga Acad Sci Biol Ser B.

[CR15] Zhao H, Star RA, Muallem S (1994). Membrane localization of H^+^ and HCO_3_^−^ transporters in the rat pancreatic duct. J Gen Physiol.

